# Synthesis and evaluation of anticancer activities of 2- or 4-substituted 3-(*N*-benzyltriazolylmethyl)-13α-oestrone derivatives

**DOI:** 10.1080/14756366.2020.1838500

**Published:** 2020-10-29

**Authors:** Rebeka Jójárt, Seyyed Ashkan Senobar Tahaei, Péter Trungel-Nagy, Zoltán Kele, Renáta Minorics, Gábor Paragi, István Zupkó, Erzsébet Mernyák

**Affiliations:** a Department of Organic Chemistry, University of Szeged, Szeged, Hungary; b Department of Pharmacodynamics and Biopharmacy, University of Szeged, Szeged, Hungary; c Department of Medicinal Chemistry, University of Szeged, Szeged, Hungary; d MTA-SZTE Biomimetic Systems Research Group, University of Szeged, Szeged, Hungary

**Keywords:** Hirao reaction, azide–alkyne cycloaddition, antiproliferative effect, tubulin polymerisation, molecular dynamics

## Abstract

2- or 4-Substituted 3-*N*-benzyltriazolylmethyl-13α-oestrone derivatives were synthesised via bromination of ring A and subsequent microwave-assisted, Pd-catalysed C(sp^2^)–P couplings. The antiproliferative activities of the newly synthesised brominated and phosphonated compounds against a panel of human cancer cell lines (A2780, MCF-7, MDA-MB 231) were investigated by means of MTT assays. The most potent compound, the 3-*N*-benzyltriazolylmethyl-4-bromo-13α-oestrone derivative exerted substantial selective cell growth-inhibitory activity against A2780 cell line with a submicromolar IC_50_ value. Computational calculations reveal strong interactions of the 4-bromo derivative with both colchicine and taxoid binding sites of tubulin. Disturbance of tubulin function has been confirmed by photometric polymerisation assay.

## 1. Introduction

**Figure 1.**
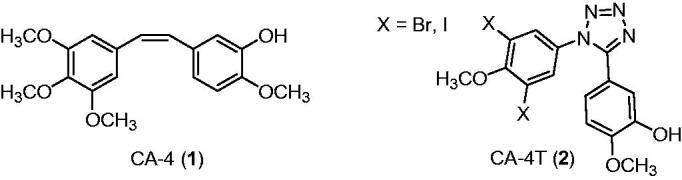
Structures of combrestatin A-4 and its tetrazolyl derivative.

The development of anticancer agents is often based on synthetic modifications of endogenous compounds^1^. However, this approach might be limited by the retained original biological activity of the biomolecule. This happens in the case of antiproliferative drug candidates based on sex hormones. Certain oestrone derivatives efficiently suppress the growth of different tumour cells, but their retained oestrogenic behaviour limits their application. Nevertheless, directed chemical modifications of the estrane core may lead to the reduction of oestrogenic action. The inversion of configuration at C-13 or opening of ring D results in core-modified oestrone derivatives with complete loss of oestrogenic activity^2–5^. Accordingly, 13α-oestrone and D-secoestrone are promising scaffolds for the development of antitumoral oestrone derivatives lacking hormonal side effects. Literature reveals certain potent anticancer oestrone derivatives, but their mechanism of action is often unclarified^1^. There exist candidates acting via inhibition of oestrogen biosynthesis; however, the majority of this compound group target other objects, including transporter proteins or tubulin. Microtubules (MTs) consist of α- and β-tubulin heterodimers that play key role in cell division^6^. Drugs that interfere with tubulin polymerisation/depolymerisation dynamics might lead to suppression of the cell growth^7–9^. Drugs that target the MT might be divided into two groups. MT destabilising agents (MDAs) prevent polymerisation of tubulin and promote depolymerisation, whereas MT stabilising agents (MSAs) promote polymerisation of tubulin and stabilise the polymer, preventing depolymerisation. There exist six binding sites on tubulin polymer^7^^,^^10^^,^^11^. MSAs, in general, bind reversibly to the taxoid binding site. Several antitubulin agents targeting vinca alkaloid or taxane sites (TBS) have been approved by Food and Drug Administration (FDA), but their application is limited due to their inefficiency against multidrug resistant (MDR) cells. On the other hand, colchicine site-binding candidates (CBS) are often still active against MDR cells, too. Combrestatin A-4 (CA-4) is a colchicine site-binding nanomolar antitubulin agent, arresting the cells in metaphase. Moreover, it is assigned as a potent vascular disrupting agent. It is of note that certain CA-4 derivatives are in clinical trials as chemotherapeutic agents. X-ray crystal structures of tubulin show that there are three zones and a bridge in this binding site. The typical colchicine site-binding agent consists of two aryl rings and a bridge, which determine the relative orientation of the rings^11^. According to literature reports, replacement of methoxy groups with halogens and introduction of a triazole or tetrazole ring instead of an ethylene bridge might be a powerful strategy in the development of more effective antitubulin CA-4 derivatives (Figure 1)^12^. The triazole heterocycle is widely used in drug development according to its favourable characteristics. It might enhance the stability against metabolic degradation and the H-bonding ability. Additionally, this heterocyclic ring is an excellent mimetic of a peptide bond^13^.

**Figure 2.**
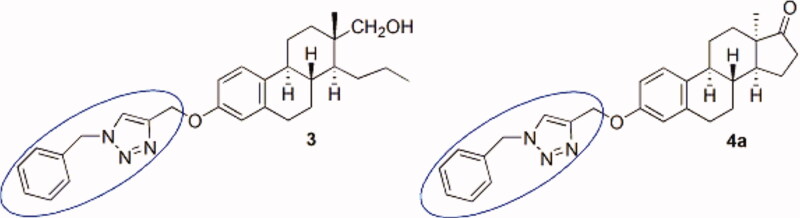
Structures of potent antiproliferative core-modified oestrone derivatives.

We have recently synthesised steroidal triazoles via the transformation of the phenolic OH group of the core-modified D-secoestrone scaffold^14^. 13α and 13β epimers of D-seco derivatives were used as starting compounds. The triazole moiety was introduced onto C-3-*O* via CuAAC reaction of 3-(prop-2-inyloxy) derivatives with benzyl azides. The evaluation of cell growth-inhibitory properties of 3-[(1-benzyl-1,2,3-triazol-4-yl)methoxy]-D-secoestrones against certain cervical, breast, and ovarian cancer cells was carried out. The determination of structure–activity relationship revealed that the antiproliferative effect greatly depends on both the orientation of the angular methyl group and the nature and size of the *para* substituent of the benzyl group. 13β Derivatives seemed to be generally more active, but a 13α compound displayed a substantial effect. The most potent compound displayed an IC_50_ value in the low micromolar range. It was proved that the presence of the phenolic OH group is disadvantageous concerning the desired antiproliferative activity, but the introduction of a benzyl or, in particular, a 1-benzyl-1,2,3-triazol-4-yl moiety onto C-3-*O* leads to marked activity improvements. D-Secoestrone triazole **3** (Figure 2) was subjected to additional biological investigations in order to shed light on its mechanism of action^15^. The immunocytochemical flow cytometric analysis alluded to a cell cycle arrest at G2/M in HeLa cells with cell accumulation in the M phase. It was proved by an *in vitro* tubulin polymerisation assay that compound **3** significantly increases the maximum rate of microtubule formation. The antimigratory experiment showed that this triazole (**3**) inhibits the migration and invasion of HeLa cells. Based on these encouraging results, the 1-benzyl-1,2,3-triazol-4-yl moiety was introduced onto C-3-*O* of 13α-oestrone bearing an intact ring D^16^. Our concept was to improve the one-micromolar IC_50_ value of the best D-secoestrone triazole by synthesising new compounds bearing the same structural element at C-3-*O*, but on the other promising, hormonally inactive 13α-oestrone scaffold. The most potent compound (**4a**) was that without any additional *para* substituent with IC_50_ values in the submicromolar range. These results highlight the importance of 13α-oestrone as a scaffold and the 3-*N*-benzyltriazolylmethyl moiety as a key element in the development of potent oestrone-based antiproliferative agents lacking oestrogenic action.

**Figure 3.**
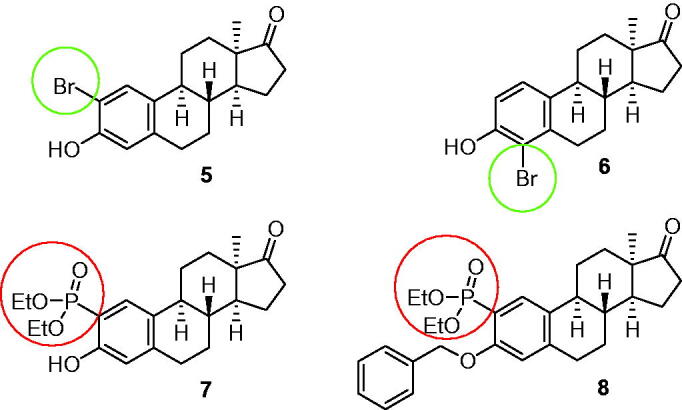
Structures of potent 17β-HSD1 and OATP2B1 inhibitors.

In recent years, we turned our attention on the synthesis of novel 2- or 4-substituted 13α-oestrone derivatives. First ring A halogenations and then Pd-catalysed C–P cross-coupling reactions were carried out^17^^,^^18^. Hirao reaction is widely used for the synthesis of arylphosphonates from aryl halides^19^. Variations of the reaction have been described under traditional thermal conditions or microwave-irradiation^20–22^. Dialkyl phosphites are usually used as the reagents, Pd(PPh_3_)_4_ as the catalyst and Et_3_N as the base. Our certain novel halo and phosphono 13α-oestrone derivatives displayed outstanding inhibitory activities against enzymes (steroid sulfatase, STS and 17β-hydroxysteroid dehydrogenase 1, 17β-HSD1) involved in oestrogen biosynthesis. Concerning oestrogen-dependent diseases, the suppression of local oestrogen production might serve as an effective therapy. This strategy might be intensified by the inhibition of polypeptides transporting organic anions (OATPs), which are able to transport oestrone-3-sulfate (E1S) into cells^23^^,^^24^. The desulphation of E1S and the stereospecific reduction of E1 result in E2 with a marked cell proliferative potential. Certain OATPs, known as E1S transporters, are overexpressed, among others, in breast and ovarian tumours. It is of note that both 2-bromo- and 4-bromo-13α-oestrone derivatives (**5** and **6**, Figure 3), synthesised recently, exerted outstanding 17β-HSD1 inhibition (IC_50_ = ∼ 1 µM). Compound **6**, however, displayed dual STS and 17β-HSD1 inhibition. Additionally, 3-hydroxy-2-phosphonate **7** proved to be dual 17β-HSD1 and OATP2B1 inhibitor with IC_50_ values of 1–2 µM, whereas its 3-benzyloxy counterpart (**8**) exhibited selective OATP2B1 inhibition with IC_50_ = 0.2 µM (Figure 3)^18^.

Based on our above-mentioned structure–activity results obtained in antiproliferative, tubulin polymerisation and OATP2B1 transport assays, our aim in the present study was to combine the key structural elements (highlighted in blue, green, and red in Figures 2 and 3) to get potent antiproliferative compounds. Here we disclose the synthesis of 3-*N*-benzyltriazolylmethyl-13α-oestrone derivatives brominated or phosphonated at C-2 or C-4.

## 2. Results

### 2.1. Chemistry

**Scheme 1.**
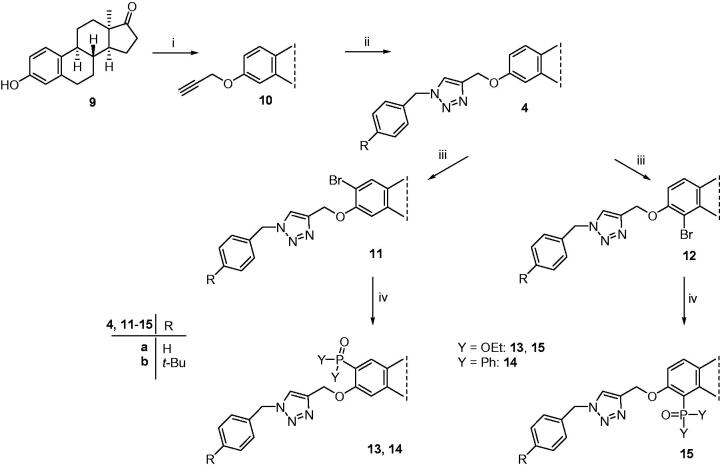
Synthesis of 2-substituted 3-*N*-benzyltriazolylmethyl-13α-oestrone derivatives.

The synthesis of 3-*N*-benzyltriazolylmethyl-13α-oestrone derivatives substituted at C-2 or C-4 was started with the propargylation of 13α-oestrone **9** (Scheme 1). The terminal alkyne function was introduced via our method established earlier^16^ using propargyl bromide as the reagent. The resulting 3-(prop-2-inyloxy) compound (**10**) was subjected to CuAAC reaction with benzyl azides differing in their *para* substituent (R = H or *t*-Bu). The click reactions afforded the desired triazolyl derivatives (**4a** and **4b**) in high yields. The next transformation was the bromination of compounds **4a** and **4b**. Electrophilic substitutions were carried out with 1 equiv. of *N*-bromosuccinimide as a brominating agent. Halogenations occurred in *ortho* positions relative to the C-3-*O* function, yielding the two regioisomers in a ratio of **11:12 **=** **2:1. Bromo derivatives (**11a**,**b** or **12a**,**b**) were subjected to Pd-catalysed reactions with diethyl phosphite or diphenylphosphine oxide as coupling partners. Microwave-assisted Hirao couplings afforded new 2- or 4-phosphonated 3-*N*-benzyltriazolylmethyl-13α-oestrone derivatives (**13**–**15**) in excellent yields. The structures of the newly synthesised bromides and phosphonates (**11**–**15**) were deduced from ^1^H and ^13 ^C NMR spectra.

### 2.2. Antiproliferative activities

The new compounds (**11**–**15**) were evaluated for their cell growth-inhibitory action against an ovarian (A2780) and two breast (MCF-7 and MDA-MB-231) human adherent cancer cell lines. As a general tendency, ovarian cell line proved to be more sensitive for the tested agents than the utilised breast cancers. Certain newly synthesised derivatives exhibited substantial sub- or low-micromolar antiproliferative potentials (Table 1). Bromo derivatives (**11** and **12**) did not influence the growth of the tumour cells, except compound **12a**, which inhibited the proliferation of A2780 cells with a submicromolar IC_50_ value. This test compound displayed substantially higher IC_50_ values against the two other cell lines. Derivatives **13b** and **14a** proved to be the most potent in the phosphonate compound group with IC_50_ values in the low micromolar range against all tested cell lines, which are comparable to those of reference agent cisplatin. Phosphonates exhibited a similar level of potency against MCF-7 and MDA-MB-231 cell lines. The only exception is compound **15 b**, which did not exert considerable growth inhibitory action against MDA-MB-231 cells. The cancer selectivity of compound **12a** was tested by means of the MTT assay using the non-cancerous mouse embryo fibroblast cell line NIH/3T3. The treatment with compound **12a** resulted in a modest inhibition of cell growth (28.73 ± 1.26% and 37.94 ± 0.75% in 10 and 30 µM, respectively) indicating the cancer selective property of the determined antiproliferative action.

**Table 1.** Antiproliferative properties of the synthesised compounds

Comp.	Conc. (µM)	Inhibition (%) ± SEM [calculated IC_50_]^a^		
		A2780	MDA- MB-231	MCF-7
**11a**	10	44.87 ± 0.09	47.49 ± 1.21	29.06 ± 1.42
	30	52.00 ± 0.80	38.18 ± 2.78	36.49 ± 1.22
		[21.51]		
**11b**	10	30.18 ± 2.35	24.39 ± 2.20	–^b^ –
	30	33.94 ± 2.70	23.92 ± 1.07	
**12a**	10	93.16 ± 0.47	52.94 ± 1.32	41.98 ± 0.97
	30	95.43 ± 0.42	53.81 ± 2.43	52.50 ± 0.94
		[0.55]	[8.80]	[12.69]
**12b**	10	46.93 ± 1.75	–	23.46 ± 1.03
	30	54.72 ± 0.70	–	29.19 ± 2.94
		[18.34]		
**13a**	10	29.53 ± 1.86	–	–
	30	90.85 ± 0.40	35.95 ± 3.17	43.54 ± 2.63
		[13.52]		
**13b**	10	95.61 ± 0.59	57.03 ± 2.58	73.99 ± 1.88
	30	99.73 ± 0.21	98.93 ± 0.20	95.52 ± 0.23
		[2.95]	[9.51]	[6.59]
**14a**	10	95.97 ± 1.28	81.95 ± 2.49	66.93 ± 1.46
	30	98.06 ± 0.89	98.52 ± 0.10	92.52 ± 1.08
		[4.87]	[7.13]	[8.38]
**14b**	10	85.52 ± 0.64	46.57 ± 1.21	77.87 ± 0.66
	30	95.82 ± 0.12	71.28 ± 1.23	90.69 ± 0.18
		[5.07]	[13.64]	[7.16]
**15a**	10	79.93 ± 1.08	25.67 ± 1.76	42.44 ± 2.94
	30	99.50 ± 0.03	96.87 ± 0.28	91.04 ± 1.49
		[5.91]	[13.15]	[11.39]
**15b**	10	46.25 ± 1.27	–	30.14 ± 1.53
	30	92.05 ± 0.86	34.79 ± 2.20	77.45 ± 1.56
		[9.96]		
Cisplatin		83.57 ± 1.21	67.51 ± 1.01	53.03 ± 2.29
		95.02 ± 0.28	87.75 ± 1.10	86.90 ± 1.24
		[1.30]	[3.70]	[5.78]

^a^ Mean value from two independent measurements with five parallel wells; standard deviation <20%.

^b^ Inhibition values <20% are not presented.

### 2.3. Tubulin polymerisation assay

**Figure 4.**
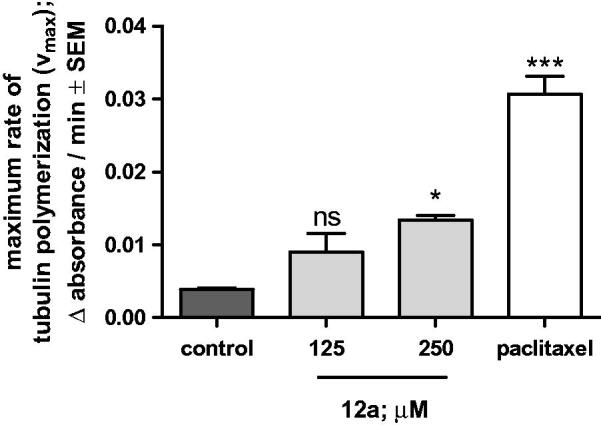
Effects of **12a** and 10 µM paclitaxel on the calculated maximum reaction rate (V_max_) of *in vitro* microtubule formation. Control: untreated samples. The experiment was performed in two parallels and the measurements were repeated twice. Each bar denotes the mean ± SEM, *n* = 4. ns, * and ***indicate *p* > 0.05, *p* < 0.05 and *p* < 0.001, respectively, compared with the control values.

Previously, D-secoestrone triazole (**3**) was proved to significantly increase maximum rate of tubulin polymerisation^15^. Based on structural similarity between compound **3** and the newly synthesised **12a** owing the lowest IC_50_ value against ovarian cancer cell line A2780, **12a** was supposed to influence microtubule formation. To demonstrate our hypothesis, **12a** was subjected to a cell-free, *in vitro* tubulin polymerisation assay in two different concentrations (125 and 250 µM). The calculated maximum rate of tubulin polymerisation was increased by our test compound which was significant when **12a** was added in 250 µM concentration to the reaction mixture (Figure 4). Paclitaxel, the positive control agent recommended by the manufacturer, evoked a threefold increase in *V*_max_ (Figure 4).

### 2.4. Computational simulations

First, docking studies have been performed for the newly synthesised most potent antiproliferative compound **12a** and for secosteroid **3** selected as a reference compound. Two potential binding sites, CBS and TBS, have been chosen on the tubulin polymer. MD investigations have been performed starting from the best docking poses of the compounds investigated. We found that the binding positions were stable for both compounds in both binding sites as they are presented by RMSD calculations for the ligands [see Figure S1(A–D) in Supplementary Materials]. Different MMGBSA binding energies collected in Table 2 clearly show that both compounds can bind to the regarded binding sites.

**Table 2.** MMGBSA binding energies (in kcal/mol) of compound **3** and **12a** in the CBS and TBS. Standard deviations of calculations are presented in parenthesis.

Compd.	CBS	TBS
**3**	−55.8 (8.3)	−58.8 (7.1)
**12a**	−63.3 (6.2)	−70.1 (6.5)

## 3. Discussion

### 3.1. Chemistry

The aim of the present work was to synthesise new 13α-oestrone derivatives as potent antiproliferative agents against human cancer cell lines of reproductive origin. Our strategy included the combination of structural elements of our promising antiproliferative or enzyme inhibitor compounds synthesised recently. Ring A was chosen as the subject for transformations and positions C-2, C-3, and C-4 were aimed to modify. Concerning the feasibility of the planned transformations, the order of the reaction steps seemed to be crucial. The activating behaviour of the phenolic OH group enables fast and effective bromination of the aromatic ring; however, the regio- and chemoselectivity is very low. To enhance the selectivity, first the 3-OH group was etherified. We have recently published that bromination of 3-*O*-methyl-13α-oestrone with 1 equiv. of NBS in dichloromethane results in a mixture of 2- and 4-bromo regioisomers in a ratio of 1:3^17^. Now we carried out the etherification of the phenolic OH group with a dual purpose: to get the two desired monobromo compounds regioselectively in the next step, and to introduce a terminal alkyne function onto C-3-*O*. We chose propargyl bromide as the reagent and performed the reaction under the conditions established earlier. The resulting phenolic ether (**10**) was suitable for the next bromination step, but the addition reactions on the terminal alkyne moiety had to be avoided. That is why we continued the sequence with the CuAAC reaction of the propargyl derivative (**10**) with two different benzyl azides (R = H or *t*-Bu). Azide reagents were selected based on the cell growth-inhibitory results of 3-*N*-benzyltriazolylmethyl-13α-oestrone derivatives synthesised and investigated earlier^16^. It has been established recently, that compound **4a** displayed outstanding antiproliferative action against certain cancer cell lines; however, its *para*-*t*-Bu counterpart **4b** did not influence cell growth markedly^16^. In this study, CuAAC reactions were performed using CuI as catalyst and PPh_3_ as an accelerating phosphine ligand. The desired triazoles (**4a** and **4b**) were formed in excellent yields. The CuAAC reactions were followed by the bromination of the 3-*N*-benzyltriazolylmethyl compounds (**4a** and **4b**) with 1 equiv. of NBS in dichloromethane. Electrophilic brominations furnished the two *ortho* regioisomers in a ratio of **8:9 **=** **2:1 in high yields. Interestingly, regioselectivity of the bromination depends markedly on the nature and size of the C-3-*O* function. The difference in regioisomeric ratios compared to those of 3-*O*-Me derivatives might be explained by the steric hindrance of a more bulky 3-*O* substituent in 3-*N*-benzyltriazolylmethyl compounds **4a** and **4b**. In the last step, the 2- and 4-bromo regioisomers were subjected to Hirao couplings. In our earlier study, microwave-assisted conditions for the transformations of 2- and 4-bromo-3-*O*-mehyl and −3-*O*-benzyl derivatives involved 10 mol% Pd(PPh_3_)_4_ as a catalyst, 1.3 equiv. of phosphite or phosphine oxide, and 3 equiv. K_2_CO_3_ in toluene^18^. The reaction time and temperature depended on the nature of the 3-*O* substituent. The transformations of 3-*O*-benzyl ethers required a more apolar solvent (toluene instead of acetonitrile) and harsher reaction conditions (150 °C, 30 min). Based on these experiences, we performed the present couplings in toluene at 150 °C, under microwave irradiation for 30 min. These conditions proved to be excellent for the effective synthesis of the desired phosphonates (**13a**,**b**; **14a**,**b**, and **15a**), except for that of a 4-bromo derivative bearing a 4′-*t*-Bu substituent (**15b**). This coupling required longer irradiation (150 °C, 1 h), which might be attributed to steric factors.

### 3.2. Determination of the antiproliferative activities

We described earlier that triazole **4a** exerted outstanding inhibitory activities against A2780 and MCF-7 cell lines in the range of IC_50_ = 0.5–0.6 µM. However, **4b**, its 4′-*t*-Bu counterpart did not have marked influence on the growth of the tested cell lines. Regarding the substantial difference in the antiproliferative potential of **4a** and **4b**, these two compounds have been selected for further transformations. Besides testing the newly synthesised compounds on A2780 (ovarian carcinoma) and MCF-7 (breast adenocarcinoma, expressing the oestrogen, progesterone, and androgen receptors), an additional cell line, the triple-negative breast carcinoma MDA-MB-231, was also included in our study. Based on the present results obtained for the phosphonates (Table 1), it can be stated, that this type of modification did not improve the high potency of parent compound **4a**. The cell growth-inhibitory potential of the phosphonates is far behind to that of unsubstituted **4a**. The low micromolar IC_50_ values of the phosphonates (**13b**, **14a**,**b**, and **15a**) reflect their moderate antiproliferative potential. Interestingly, phosphonates influenced the growth of A2780 cells most. Considering the two breast cancer cell lines with different receptorial status, no significant difference in growth-inhibitory activities have been observed. However, two compounds (**12a** and **14a**) proved to be more potent against the triple-negative MDA-MB-231 line. The presented pharmacological results are considered preliminary and, therefore, no conclusion can be made concerning the mechanism of the action. However, based on the comparison of the IC_50_ values obtained on the two breast cancer cell lines, a receptor-independent mechanism could be proposed. Results obtained for the 2-bromo compounds (**11a**,**b**) reveal that bromination at this position is disadvantageous concerning the antiproliferative potential against the tested cell lines. However, the other regioisomer without the 4′-*t*-Bu group (**12a**), proved to be highly potent with selective action against A2780 cells. The dependence of the cell growth-inhibitory potential on the regioisomerism is a very important structure–activity result. Interestingly, the empirical rules established earlier proved to be valid for the bromo derivatives (**11a**,**b** and **12a**,**b**) as well. The presence of the 4′-*t*-Bu group on the newly introduced benzylic moiety was also detrimental.

The cancer selectivity of compound **12a** was tested by means of the MTT assay using the non-cancerous mouse embryo fibroblast cell line NIH/3T3. The growth inhibitory effect was found to be substantially lower than those against cancer cell lines. Since the inhibition of proliferation was less than 40% even at the highest concentration (30 µM), the IC_50_ value was not calculated but it is definitely above 30 µM. This kind of viability assay cannot be considered to be sufficient to declare a cancer-selective action. The huge difference in the determined antiproliferative properties may reflect a cell type-dependent action instead of a general toxic character indicating the relevance of the presented structure in lead-finding projects.

### 3.3. Tubulin polymerisation assay

Performing a 60-min-long tubulin polymerisation assay a direct effect of **12a** has been demonstrated on microtubule formation. The significant increase in *V*_max_ induced by our test compound is similar to the effects of other oestrone derivatives from D-secoalcohol^25^ and D-secoestrone-triazole^15^ series. However, another ring A substituted cytotoxic oestradiol analogue, 2-methoxyestradiol, has been reported to inhibit tubulin polymerisation^26^. This result is suitable for providing evidence about the final effect of our test compound on tubulin-microtubule system.

### 3.4. Computational simulations

**Figure 5.**
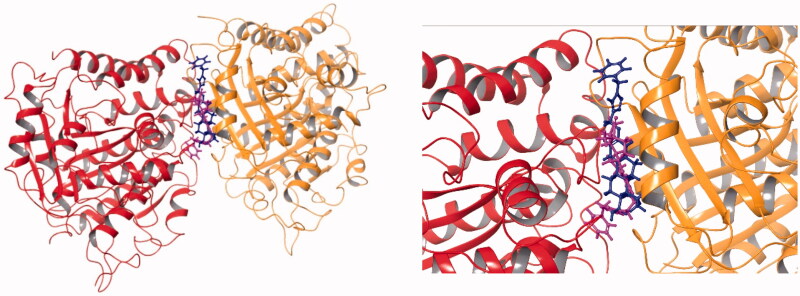
Best docking poses of compound **3** and **12a** in the CBS of tubulin dimer. The dark blue structure represents compound **3**, while purple marks compound **12a**.

**Figure 6.**
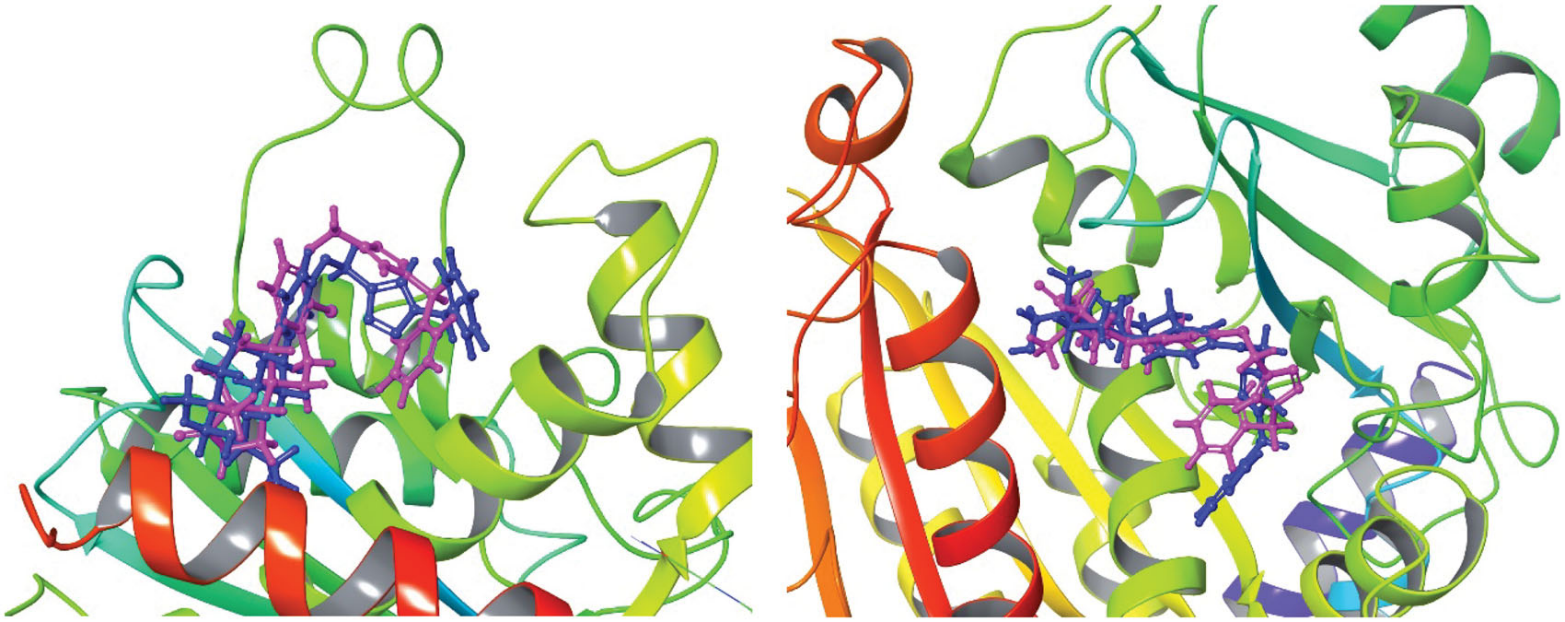
Best docking poses of compound **3** and **12a** in the TBS of tubulin monomer. The dark blue structure represents compound **3** while purple marks compound **12a**.

We have demonstrated earlier that core-modified oestrone derivative **3** might be considered as an MSA. However, the majority of antitubulin oestrone derivatives described in literature belong to the MDA group, acting at the CBS of tubulin. From the comparison of the structures of the brominated combrestatine triazole **2** as an MDA and oestrone derivative **3**, it can be stated that they possess similar structural elements, such as the two aryl systems connected with a tetrazole or triazole bridge. It was shown by Beale et al. that the presence of bromines in compound **2** is advantageous concerning the antitubulin action. Interestingly, the two compounds belong to different MT targeting groups. Here we synthesised a new compound (**12a**) with structural similarity to both MT targeting agents **2** and **3**. Based on these structural similarities and the substantial antiproliferative action of new derivative **12a**, here we performed computational studies to investigate the possible interaction of this compound with tubulin. Our selection, concerning the potential binding region of compound **12a** out of the known 6 possibilities^7^^,^^10^ taking tubulin surface, was based on the following considerations. (i) Oestrone derivatives usually interact with tubulin at the CBS^11^. (ii) Ligands which promote polymerisation of tubulin usually bind to the TBS^7^^,^^8^. Because we did not have experimental evidence for the exact binding position of compound **12a**, both potential binding sites (CBS and TBS) were considered. Secosteroid **3** was selected as a reference compound and, altogether, four different complexes were investigated in the simulations. Molecular docking studies were performed first in order to get the best poses for the following MD calculations. In Figures 5 and 6, we represented the binding poses of ligands **3** and **12a** in CBS and TBS, respectively. The purple structure always represents compound **12a**, while secoestrone **3** is represented in dark blue. It is clear, that in the TBS both compounds **3** and **12a** adopted almost the same binding position, while in the CBS the estrane cores occupied a common region, but in a reverse manner. Consequently, the 3-*N*-benzyltriazolylmethyl moiety oriented in an opposite way in the two cases.

Concerning binding preference order, SP docking score only helps to separate binding and non-binding molecules in a molecular pocket. However, it is not suitable to determine an accurate binding preference order; therefore, molecular dynamics (MD) calculations were performed. This allowed us to calculate binding energy at a more advanced level (MMGBSA method). Furthermore, calculations also provide information about the stability of the binding pose concerning the different ligand–protein complexes. It was established that the binding positions were stable in all four cases, even though the two compounds occupy the CBS in reversed manner (Table 2). Comparing binding energies at the same region, compound **12a** had always stronger interaction than compound **3**. Comparing binding energies at the different binding sites, compound **3** provided almost the same interaction energies in the two binding pockets, while compound **12a** had stronger interaction at the TBS. The strong interactions of compound **12a** indicate that the hormonally inactive 13α-estrane core with certain ring A modifications might be a suitable scaffold in the design of potent MT targeting agents. Concerning its possible dual binding (at CBS and at TBS), it might be a promising candidate in the development of antitubulin drugs targeting MDR cells, too.

## 4. Materials and methods

### 4.1. Chemistry

Melting points (Mp) were determined with a Kofler hot-stage apparatus and are uncorrected. Elemental analyses were performed with a Perkin-Elmer CHN analyser model 2400 (PerkinElmer, Waltham, MA). Thin-layer chromatography: silica gel 60 F254; layer thickness 0.2 mm (Merck); eluents (ss): A: 50% ethyl acetate/50% hexane, B: ethyl acetate, C: 2% methanol/98% ethyl acetate, detection with I_2_ or UV (365 nm) after spraying with 5% phosphomolybdic acid in 50% aqueous phosphoric acid and heating at 100–120 °C for 10 min. Flash chromatography: silica gel 60, 40–63 µm (Merck, Kenilworth, NJ). Reactions under microwave irradiation were carried out with a CEM Corporation focussed microwave system, Model Discover SP. The maximum power of irradiation was 200 W. ^1^H NMR spectra were recorded in DMSO-d_6_, CDCl_3_ solution with a Bruker DRX-500 instrument (Bruker, Billerica, MA) at 500 MHz, with Me_4_Si as internal standard. ^13 ^C NMR spectra were recorded with the same instrument at 125 MHz under the same conditions. Mass spectrometry: full scan mass spectra of the compounds were acquired in the range of 50–1000 *m*/*z* with a Finnigan TSQ-7000 triple quadrupole mass spectrometer (Finnigan-MAT, San Jose, CA) equipped with a Finnigan electrospray ionisation source. Analyses were performed in positive ion mode using flow injection mass spectrometry with a mobile phase of 50% aqueous acetonitrile containing 0.1 v/v% formic acid. The flow rate was 0.3 ml/min. Five µl aliquot of the samples were loaded into the flow. The ESI capillary was adjusted to 4.5 kV and N_2_ was used as a nebuliser gas.

#### 4.1.1. Synthesis of 3-(prop-2-inyloxy)-13α-estra-1,3,5(10)-triene (10)

3-Hydroxy-13α-estra-1,3,5(**10**)-trien-17-one (**1**, 540 mg, 2.0 mmol) was dissolved in acetone (15 ml), then propargyl bromide [0.34 ml (80 wt.% in toluene), 3.0 mmol], and K_2_CO_3_ (1.94 g, 14 mmol) were added. The reaction mixture was stirred at 70 °C for 24 h, the solvent was then evaporated off, and the residue was purified by flash chromatography with EtOAc/CH_2_Cl_2_ = 2/98 as eluent. Compound **7** was obtained as a white solid (610 mg, 98%), mp 133‒134 °C, R_f_ = 0.70 (ss B); Anal calcd. for C_21_H_24_O_2_: C, 81.78; H, 7.84. Found: C, 81.93; H, 7.64. ^1^H NMR: *δ* ppm H 1.06 (s, 3H, H-18); 2.49 (s, 1H, C≡CH); 2.83 (m, 2H, H-6); 4.65 (s, 2H, OCH_2_); 6.68 (s, 1H, H-4); 6.77 (d, *J* = 8.5 Hz, 1H, H-2); 7.19 (d, *J* = 8.5 Hz, 1H, H-1). Compound **7** is identical with the compound described in Ref. [16].

#### 4.1.2. Synthesis of 3-[{1-benzyl-1*H*-1,2,3-triazol-4-yl}methoxy]- and 3-[{1-(4-*tert*-butylbenzyl)-1*H*-1,2,3-triazol-4-yl}methoxy]-13α-estra-1,3,5(10)-trien-17-one (4a and 4 b)

To a stirred solution of 3-(prop-2-inyloxy)-13α-estra-1,3,5(10)-trien-17-one **7** (616 mg, 2.0 mmol) in toluene (8 ml), Ph_3_P (52 mg, 0.2 mmol), CuI (19.0 mg, 0.1 mmol), DIPEA (1.04 ml, 6.0 mmol), and benzylazide or 4-*tert*-butyl-benzylazide (1 equiv.^16^) were added. The reaction mixtures were refluxed for 2 h, cooled to rt and evaporated *in vacuo*. The residues were purified by flash chromatography with EtOAc/CH_2_Cl_2_ = 5/95 as eluent. Compound **4a** was obtained as a white solid (862 mg, 97%), mp 164‒165 °C, R_f_ = 0.35 (ss C); ^1^H NMR: *δ* ppm 1.05 (s, 3H, H-18); 2.80 (m, 2H, H-6); 5.14 (s, 2H, OCH_2_); 5.51 (s, 2H, NCH_2_); 6.67 (s, 1H, H-4); 6.75 (dd, *J* = 8.5 Hz, *J* = 2.0 Hz, 1H, H-2); 7.16 (d, *J* = 8.5 Hz, 1H, H-1); 7.27 (m, 2H, H-2′, H-6′); 7.36 (m, 3H, H-3′, H-4′, H-5′); 7.50 (s, 1H, C = CH). Compound **4a** is identical with the compound described in Ref. [16].

Compound **4b** was obtained as a white solid (961 mg, 96%), mp 111‒112 °C, R_f_ = 0.28 (ss C); ^1^H NMR: *δ* ppm 1.05 (s, 3H, H-18); 1.31 (s, 3 × 3H, C(CH_3_)3); 2.81 (m, 2H, H-6); 5.11 (s, 2H, OCH_2_); 5.50 (s, 2H, NCH_2_); 6.69 (s, 1H, H-4); 6.78 (m, 1H, H-2); 7.16‒7.20 (overlapping multiplets, 3H, H-1, H-2′, H-6′); 7.39 (d, 2H, H-3′, H-5′); 7.55 (s, 1H, C = CH). Compound **4a** is identical with the compound described in Ref. [16].

#### 4.1.3. General procedure for the bromination of triazoles 4a and 4b

Triazole **4a** or **4b** (442 mg or 498 mg, 1.00 mmol) was dissolved in dichloromethane (5 ml) and NBS (178 mg, 1.00 mmol) was added. The mixture was stirred at rt for 2.5 h, the solvent was then evaporated off and the crude product was purified by flash chromatography with EtOAc/hexane = 30/70 as eluent.

##### 4.1.3.1. Synthesis of 3-[{1-benzyl-1*H*-1,2,3-triazol-4-yl}methoxy]-2-bromo-13α-estra-1,3,5(10)-trien-17-one (11a) and 3-[{1-benzyl-1*H*-1,2,3-triazol-4-yl}methoxy]-4-bromo-13α-estra-1,3,5(10)-trien-17-one (12a)

The first-eluting **12a** was obtained as a white solid (160 mg, 31%). Mp.: 188–190 °C. R_f_=0.66 (ss A). Anal calcd. for C_28_H_30_BrN_3_O_2_: C, 64.26; H, 5.81. Found: C, 64.34; H, 5.89. ^1^H NMR (CDCl_3_) δ ppm: 1.05 (s, 3H, H-18), 2.65 and 3.00 (2 × m, 2 × 1H, H-6), 5.24 (m, 2H, OCH_2_), 5.52 (s, 2H, NCH_2_), 6.87 (d, *J* = 8.6 Hz, 1H, H-2), 7.19 (d, *J* = 8.6, 1H, H-1), 7.27–7.28 (overlapping multiplets, 2H, H-2′ and H-6′), 7.34–7.37 (overlapping multiplets, 3H, H-3′, H-4′ and H-6′), 7.58 (s, 1H, C  =  CH). ^13 ^C NMR (CDCl_3_) *δ* ppm: 21.0 (CH_2_), 25.0 (C-18), 28.3 (CH_2_), 28.4 (CH_2_), 31.6 (CH_2_), 31.9 (CH_2_), 33.4 (CH_2_), 40.6 (CH), 41.7 (CH), 49.0 (CH), 50.0 (C-13), 54.2 (NCH_2_), 63.7 (OCH_2_), 111.4 (C-2), 115.2 (C-4), 122.7 (C  =  CH), 125.5 (C-1), 128.0 (2 C, C-3′ and C-5′), 128.8 (C-4′), 129.1 (2 C, C-2′ and C-6′), 134.5 (C-1′), 134.9 (C-10), 137.9 (C-5), 144.7 (C  =  CH),152.6 (C-3), 221.4 (C-17). MS: [M + H]^+^ (79/81Br) 519 and 521.

The next-eluting **11a** was obtained as a white solid (319 mg, 61%). Mp.: 151–154 °C. R_f_=0.54 (ss A). Anal calcd. for C_28_H_30_BrN_3_O_2_: C, 64.26; H, 5.81. Found: 64.36; H, 5.88. ^1^H NMR (CDCl_3_) δ ppm: 1.05 (s, 3H, H-18), 2.70–2.82 (overlapping multiplets, 2H, H-6), 5.26 (m, 2H, OCH_2_), 5.55 (s, 2H, NCH_2_), 6.72 (s, 1H, H-4), 7.27–7.29 (overlapping multiplets, 2H, H-2′, and H-6′), 7.38–7.39 (overlapping multiplets, 3H, H-3′, H-4′, H-6′), 7.66 (s, 1H, C  =  CH). ^13 ^C NMR (CDCl_3_) *δ* ppm: 20.9 (CH_2_), 25.0 (C-18), 28.0 (CH_2_), 28.2 (CH_2_), 30.0 (CH_2_), 31.9 (CH_2_), 33.4 (CH_2_), 41.1 (CH), 41.3 (CH), 49.1 (CH), 50.1 (C-13), 54.8 (NCH_2_), 63.1 (OCH_2_), 109.4 (C-2), 114.3 (C-4), 123.2 (C=CH), 128.2 (2 C, C-3′, and C-5′), 129.1 (C-4′), 129.2 (2 C, C-2′, and C-6′), 130.8 (C-1), 133.8 (C-10), 134.7 (C-1′), 137.6 (C-5), 144.1 (C=CH), 152.1 (C-3), 221.4 (C-17). MS *m*/*z* (%): MS: [M + H]^+^ (79/81Br) 519 and 521.

##### 4.1.3.2. Synthesis of 2-bromo-3-[{1–(4-*tert*-butylbenzyl)-1*H*-1,2,3-triazol-4-yl}methoxy]-13α-estra-1,3,5(10)-trien-17-one (11 b) and 4-bromo-3-[{1–(4-*tert*-butylbenzyl)-1*H*-1,2,3-triazol-4-yl}methoxy]-13α-estra-1,3,5(10)-trien-17-one (12 b)

The first-eluting **12 b** was obtained as a white solid (98 mg, 17%). Mp.: 178–180 °C. R_f_=0.71 (ss A). Anal calcd. for C_32_H_38_BrN_3_O_2_: C, 66.66; H, 6.64. Found: 66.73; H, 6.72. ^1^H NMR (CDCl_3_) *δ* ppm: 1.05 (s, 3H, H-18), 1.31 (s, 9H, 4′-C(CH_3_)_3_), 2.65 and 3.00 (2 × m, 2 × 1H, H-6), 5.23 (m, 2H, OCH_2_), 5.49 (s, 2H, NCH_2_), 6.87 (d, *J* = 8.7 Hz, H-2), 7.18 (d, *J* = 8.7 Hz, 1H, H-1), 7.20 (d, *J* = 8.4 Hz, 2H, H-2′, and H-6′), 7.38 (d, *J* = 8.4 Hz, 2H, H-3′, and H-5′), 7.58 (s, 1H, C = CH). ^13 ^C NMR (CDCl_3_) *δ* ppm: 21.0 (CH_2_), 25.0 (C-18), 28.3 (CH_2_), 28.4 (CH_2_), 31.2 (3 C, 4′-C(CH_3_)_3_), 31.6 (CH_2_), 31.9 (CH_2_), 33.4 (CH_2_), 34.6 (4′-C(CH_3_)_3_), 40.6 (CH), 41.7 (CH), 49.0 (CH), 50.0 (C-13), 53.9 (NCH_2_), 63.6 (OCH_2_), 111.4 (C-2), 115.2 (C-4), 122.6 (C=CH), 125.5 (C-1), 126.0 (2 C, C-3′, and C-5′), 127.8 (2 C, C-2′, and C-6′), 131.5 (C-10), 134.9 (C-1′), 137.9 (C-5), 144.6 (C=CH), 151.9 and 152.6 (2 C, C-3, and C-4′), 221.4 (C-17). MS: [M + H]^+^ (79/81Br) 575 and 577. Continued elution yielded first a mixture of **12 b** (80 mg, 14%) and **11 b** (140 mg, 24%), and then compound **11 b** (218 mg, 38%) as a white solid. Mp.: 148–150 °C. R_f_=0.62 (ss A). Anal calcd. for C_32_H_38_BrN_3_O_2_: C, 66.66; H, 6.64. Found: 64.72; H, 6.72. ^1^H NMR (CDCl_3_) *δ* ppm: 1.05 (s, 3H, H-18), 1.37 (s, 9H, 4′-C(CH_3_)_3_), 2.70–2.82 (overlapping multiplets 2H, H-6), 5.22 (m, 2H, OCH_2_), 5.49 (s, 2H, NCH_2_), 6.74 (s, 1H, H-4), 7.20 (d, *J* = 8.4 Hz, 2H, H-2′, and H-6′), 7.37–7.39 (overlapping multiplets, 3H, H-3′, H-5′, and H-1), 7.58 (s, 1H, C = CH). ^13 ^C NMR (CDCl_3_) *δ* ppm: 20.9 (CH_2_), 25.0 (C-18), 28.0 (CH_2_), 28.2 (CH_2_), 30.0 (CH_2_), 31.2 (4′-C(CH_3_)_3_), 31.9 (CH_2_), 33.4 (C), 34.6 (4′-C(CH_3_)_3_), 41.1 (CH), 41.3 (CH), 49.1 (CH), 50.0 (C-13), 53.9 (NCH_2_), 63.7 (OCH_2_), 109.5 (C-2), 114.3 (C-4), 122.6 (C=CH), 126.0 (2 C, C-3′, and C-5′), 127.8 (2 C, C-2′, and C-6′), 130.7 (C-1), 131.4 (C-10), 134.4 (C-1′), 137.4 (C-5), 144.6 (C=CH), 151.9 and 152.4 (2 C, C-3, and C-4′), 221.3 (C-17). MS: [M + H]^+^ (79/81Br) 575 and 577.

#### 4.1.4. General procedure for Hirao coupling of brominated triazoles (11a,b and 12a,b)

2- or 4-Bromo triazoles (**11a**,**b** or **12a**,**b**; 260 mg or 288 mg, 0.50 mmol), tetrakis(triphenylphosphine)palladium(0) (57.8 mg, 0.050 mmol, 10 mol%), potassium carbonate (104 mg, 0.75 mmol, 1.5 equiv.), diethyl phosphite (0.50 mmol, 69 mg) or diphenylphosphine oxide (0.50 mmol, 101 mg), and acetonitrile or toluene (5 ml) were added into a 10 ml Pyrex pressure vessel (CEM, Part #: 908035) with silicone cap (CEM, Part #: 909210). The mixture was irradiated in a CEM microwave reactor at 150 °C 30–60 min under stirring. The solvent was evaporated *in vacuo* and the residue was purified by flash chromatography.

##### 4.1.4.1. Synthesis of (3-[{1-benzyl-1H-1,2,3-triazol-4-yl}methoxy]-13α-estra-1,3,5(10)-trien-17-on-2-yl)-diethylphosphonate

The residue was purified by flash chromatography with MeOH/EtOAc = 2/98 as eluent. Compound **13a** was isolated as a white solid (84%). Mp.: 75–80 °C. R_f_=0.31 (ss B). Anal calcd. for C_32_H_40_N_3_O_5_P: C, 66.54; H, 6.98. Found: 66.62; H, 7.07. ^1^H NMR (CDCl_3_) *δ* ppm: 1.05 (s, 3H, H-18), 1.16 (t, *J* = 7.1 Hz, 6H, 2 × OCH_2_CH_3_), 2.85 (m, 2H, H-6), 3.93–4.03 (overlapping multiplets, 4H, 2 × OCH_2_CH_3_), 5.25 (m, 2H, OCH_2_), 5.53 (s, 2H, NCH_2_), 6.73 (d, *J* = 6.8 Hz, 1H, H-4), 7.27 (m. 2H, H-2′, and H-6′), 7.39 (overlapping multiplets, 3H, H-3′, H-4’and H-5′), 7.66 (d, *J* = 15.7 Hz, H-1), 7.83 (s, 1H, C = CH). ^13 ^C NMR (CDCl_3_) *δ* ppm: 16.3 (d, *J* = 6.3 Hz, 2 C: 2 × OCH_2_CH_3_), 20.9 (CH_2_), 25.0 (C-18), 27.8 (CH_2_), 28.2 (CH_2_), 30.6 (CH_2_), 31.8 (CH_2_), 33.3 (CH_2_), 41.2 (CH), 41.3 (CH), 49.1 (CH), 50.1 (C-13), 54.3 (NCH_2_), 61.9 (2 C, 2 × OCH_2_CH_3_), 63.1 (OCH_2_), 112.8 (d, *J* = 9.9 Hz, C-4), 114.1 (d, *J* = 188.9 Hz, C-2), 123.1 (C=CH), 128.1 (2 C, C-3′, and C-5′), 128.7 (C-4′), 129.1 (2 C, C-2′, and C-6′), 132.6 (d, *J* = 13.8 Hz, C-10), 132.8 (d, *J* = 8.1 Hz, C-1), 134.5 (C-1′), 144.0 (2 C, C-5, and C=CH), 157.4 (C-3), 221.4 (C-17). ^31 ^P NMR *δ* ppm: 17.8. MS *m*/*z* (%): 578 (100, [M + H]^+^).

##### 4.1.4.2. Synthesis of (3-[{1–(4-tert-butylbenzyl)-1H-1,2,3-triazol-4-yl}methoxy]-13α-estra-1,3,5(10)-trien-17-on-2-yl)-diethylphosphonate

The residue was purified by flash chromatography with MeOH/EtOAc = 2/98 as eluent. Compound **13b** was isolated as a colourless oil (83%). R_f_=0.55 (ss B). Anal calcd. for C_36_H_48_N_3_O_5_P: C, 68.23; H, 7.63. Found: 68.31; H, 7.72. ^1^H NMR (CDCl3) *δ* ppm: 1.05 (s, 3H, H-18), 1.15 (t, *J* = 7.1 Hz, 6H, 2 × OCH_2_CH_3_), 1.29 (s, 9H, 4′-C(CH_3_)_3_), 2.85 (m, 2H, H-6), 3.92–4.03 (overlapping multiplets, 4H, 2 × OCH_2_CH_3_), 5.23 (m, 2H, OCH_2_), 5.48 (s, 2H, NCH_2_), 6.73 (d, *J* = 6.9 Hz, 1H, H-4), 7.21 (d, *J* = 8.4 Hz, 2H, H-2′, and H-6′), 7.37 (d, *J* = 8.4 Hz, 2H, H-3′, and H-5′), 7.67 (d, *J* = 15.7 Hz, 1H, H-1), 7.77 (s, 1H, C = CH). ^13 ^C NMR (CDCl_3_) *δ* (ppm): 16.3 (d, *J* = 6.6 Hz, 2 C, 2 × OCH_2_CH_3_), 20.9 (CH_2_), 25.0 (C-18), 27.8 (CH_2_), 28.2 (CH_2_), 30.6 (CH_2_), 31.2 (3 C, 4′-C(CH_3_)_3_), 31.9 (CH_2_), 33.3 (CH_2_), 34.6 (4′-C(CH_3_)_3_), 41.2 (CH), 41.3 (CH), 49.1 (CH), 50.0 (C-13), 53.9 (NCH_2_), 61.8 (2 C, 2 × OCH_2_CH_3_), 63.2 (OCH_2_), 112.7 (d, *J* = 9.8 Hz, C-4), 114.1 (d, *J* = 189.4 Hz, C-2), 122.8 (C=CH), 125.9 (2 C, C-3′, and C-5′), 127.8 (2 C, C-2′, and C-6′), 131.6 (C-1′), 132.6 (d, *J* = 14.1 Hz, C-10), 132.9 (d, *J* = 7.9 Hz, C-1), 143.9 and 144.9 (2 C, C-5, and C=CH), 151.8 (C-4′), 157.5 (C-3), 221.3 (C-17). ^31 ^P NMR *δ* ppm 17.8. MS m/z (%): 634 (100, [M + H]^+^).

##### 4.1.4.3. Synthesis of (3-[{1-benzyl-1H-1,2,3-triazol-4-yl}methoxy]-13α-estra-1,3,5(10)-trien-17-on-2-yl)diphenylphosphine oxide

The residue was purified by flash chromatography with MeOH/EtOAc = 2/98 as eluent. Compound **14a** was isolated as a white solid (79%). Mp.: 117–120 °C. R_f_=0.28 (ss C). Anal calcd. for C_40_H_40_N_3_O_3_P: C, 74.86; H, 6.28. Found: 74.93; H, 6.33. ^1^H NMR (CDCl_3_) *δ* ppm: 1.03 (s, 3H, H-18), 2.87 (m, 2H, H-6), 5.00 (m, 2H, OCH_2_), 5.38 (s, 2H, NCH_2_), 6.72–6.73 (overlapping multiplets, 2H), 7.15–7.17 (overlapping multiplets, 2H), 7.24–7.30 (m, 2H), 7.34–7.41 (overlapping multiplets, 6H), 7.55–7.63 (overlapping multiplets, 6H). ^13 ^C NMR (CDCl_3_) *δ* ppm: 20.9 (CH_2_), 25.0 (C-18), 27.9 (CH_2_), 28.1 (CH_2_), 30.7 (CH_2_), 31.8 (CH_2_), 33.4 (CH_2_), 41.3 (CH), 41.5 (CH), 49.2 (CH), 50.1 (C-13), 54.0 (NCH_2_), 62.5 (OCH_2_), 112.4 (d, *J* = 6.9 Hz, C-4), 117.6 (d, *J* = 105.5 Hz, C-2), 122.6 (C=CH), 127.9 (2 C, C-3′, and C-5′), 128.0–128.2 (overlapping multiplets, 4 C), 128.7 (C-4′), 129.1 (2 C, C-2′, and C-6′), 131.3 (m, 2 C, C-4” , and C-4′”), 131.6–131.8 (overlapping multiplets, 4 C), 132.6 (C), 132.9 (d, *J* = 7.5 Hz, C-1), 133.0 (C), 133.5 (C), 134.7 (C), 143.9 (C), 144.1 (C), 157.1 (C-3), 221.2 (C-17). ^31 ^P NMR *δ* ppm: 27.2. MS *m*/*z* (%): 642 (100, [M + H]^+^).

##### 4.1.4.4. Synthesis of (3-[{1–(4-tert-butylbenzyl)-1H-1,2,3-triazol-4-yl}methoxy]-13α-estra-1,3,5(10)-trien-17-on-2-yl)diphenylphosphine oxide

The residue was purified by flash chromatography with MeOH/EtOAc = 2/98 as an eluent. Compound **14b** was isolated as a white solid (73%). Mp.: 205–208 °C. R_f_=0.42 (ss C). Anal calcd. for C_44_H_48_N_3_O_3_P: C, 75.73; H, 6.93. Found: 75.79; H, 6.99. ^1^H NMR (CDCl_3_) *δ* ppm: 1.03 (s, 3H, H-18), 1.31 (s, 9H, 4′-C(CH_3_)_3_), 2.87 (m, 2H, H-6), 5.00 (d, *J* = 4.0 Hz, 2H, OCH_2_), 5.38 (s, 2H, NCH_2_), 6.61 (s, 1H, C = CH), 6.71 (d, *J* = 5.5 Hz, 1H, H-4), 7.09 (d, 2H), 7.21 (m, 2H), 7.27 (m, 2H), 7.33–7.40 (overlapping multiplets, 4H), 7.54–7.62 (overlapping multiplets, 4H), 7.64 (d, *J* = 14.2 Hz, 1H, H-1). ^13 ^C NMR (CDCl_3_) *δ* ppm: 20.9 (CH_2_), 25.0 (C-18), 27.9 (CH_2_), 28.1 (CH_2_), 30.7 (CH_2_), 31.2 (3 C, 4′-C(CH_3_)_3_), 31.8 (CH_2_), 33.4 (CH_2_), 34.6 (4′-C(CH_3_)_3_), 41.3 (CH), 41.4 (CH), 49.1 (CH), 50.1 (C-13), 53.7 (NCH_2_), 62.3 (OCH_2_), 112.2 (d, *J* = 7.1 Hz, C-4), 117.5 (d, *J* = 105.6 Hz, C-2), 122.4 (C=CH), 125.9 (2 C, C-3′, and C-5′), 127.7 (2 C, C-2′, and C-6′), 127.9–128.1 (overlapping multiplets, 4 C), 131.2 and 131.3 (C-4” and C-4′”), 131.6–131.8 (overlapping multiplets, 4 C), 131.6–134.0 (overlapping multiplets, 4 C), 132.9 (d, *J* = 7.6 Hz, C-1), 143.9 and 144.0 (C-5 and C=CH), 151.9 (C-4′), 156.9 (d, *J* = 3.0 Hz, C-3), 221.4 (C-17). ^31 ^P NMR *δ* ppm: 26.9. MS *m*/*z* (%): 698 (100, [M + H]^+^).

##### 4.1.4.5. Synthesis of (3-[{1-benzyl-1H-1,2,3-triazol-4-yl}methoxy]-13α-estra-1,3,5(10)-trien-17-on-4-yl)-diethylphosphonate

The residue was purified by flash chromatography with MeOH/EtOAc = 2/98 as an eluent. Compound **15a** was isolated as a white solid (72%). Mp.: 43–45 °C. R_f_=0.45 (ss B). Anal calcd. for C_32_H_40_N_3_O_5_P: C, 66.54; H, 6.98. Found: C, 66.62; H, 7.07. ^1^H NMR (CDCl_3_) *δ* (ppm): 1.05 (s, 3H, H-18), 1.13 (t, *J* = 7.2 Hz, 6H, 2 × OCH_2_CH_3_), 3.26 (m, 2H, H-6), 3.88–4.01 (overlapping multiplets, 4H, 2 × OCH_2_CH_3_), 5.21 (d, *J* = 3.8 Hz, 2H, OCH_2_), 5.53 (s, 2H, NCH_2_), 6.87 (dd, *J* = 6.7 Hz, *J* = 8.4 Hz, 1H, H-2), 7.26–7.28 (m, 2H, H-2′, and H-6′), 7.33–7.38 (overlapping multiplets, 2H, H-3′, H-4′, H-5′, and H-1), 7.72 (s, 1H, C = CH). ^13 ^C NMR (CDCl_3_) δ (ppm): 16.2 (2 C, 2 × OCH_2_CH_3_), 20.9 (CH_2_), 24.9 (C-18), 28.2 (CH_2_), 28.6 (CH_2_), 29.3 (CH_2_), 32.0 (CH_2_), 33.3 (CH_2_), 40.3 (CH), 41.7 (CH), 49.5 (CH), 50.1 (C-13), 54.2 (NCH_2_), 61.3 (d, *J* = 5.2 Hz, OCH_2_CH_3_), 61.4 (d, *J* = 5.2 Hz, OCH_2_CH_3_), 63.7 (OCH_2_), 110.9 (d, *J* = 10.1 Hz, C-2), 115.1 (d, *J* = 182.0 Hz, C-4), 122.8 (C=CH), 128.1 (2 C, C-3′, and C-5′), 128.7 (C-4′), 129.1 (2 C, C-2′, and C-6′), 131.5 (C-1), 134.4 (d, *J* = 14.0 Hz, C-10), 134.6 (C-1′), 144.8 (d, *J* = 10.0 Hz, C-5), 144.8 (C=CH), 145.0 (C), 159.0 (C-3), 221.6 (C-17). ^31 ^P NMR δ (ppm): 18.2. MS *m*/*z* (%): 578 (100, [M + H]^+^).

##### 4.1.4.6. Synthesis of (3-[{1–(4-tert-butylbenzyl)-1H-1,2,3-triazol-4-yl}methoxy]-13α-estra-1,3,5(10)-trien-17-on-4-yl)-diethylphosphonate

The residue was purified by flash chromatography with EtOAc as an eluent. Compound **15b** was obtained as a white solid (70%). Mp.: 54–59 °C. R_f_=0.51 (ss B). Anal calcd. for C_36_H_48_N_3_O_5_P: C, 68.26; H, 7.63. Found: C, 68.34; H, 7.69. ^1^H NMR (CDCl_3_) *δ* (ppm) 1.05 (s, 3H, H-18), 1.11 (t, *J* = 7.1 Hz, 6H, 2 × OCH_2_CH_3_), 1.29 (s, 9H, 4′-C(CH_3_)_3_), 3.25 (m, 2H, H-6), 3.88–4.01 (overlapping multiplets, 4H, 2 × OCH_2_CH_3_), 5.20 (d, *J* = 3.9 Hz, 2H, OCH_2_), 5.49 (s, 2H, NCH_2_), 6.87 (dd, *J* = 6.6 Hz, *J* = 8.6 Hz, 1H, 2-H), 7.21 (d, *J* = 8.2 Hz, 2H, H-3′, and H-5′), 7.37 (overlapping multiplets, 3H, H-2′, H-6′, and H-1), 7.71 (s, 1H, C = CH). ^13 ^C NMR (CDCl_3_) δ (ppm): 16.2 (d, *J* = 6.6 Hz, 2 C, 2 × OCH_2_CH_3_), 20.9 (CH_2_), 24.9 (C-18), 28.1 (CH_2_), 28.6 (CH_2_), 29.3 (CH_2_), 31.2 (3 C, 4′-C(CH_3_)_3_), 31.9 (CH_2_), 33.3 (CH_2_), 34.6 (4′-C(CH_3_)_3_), 40.3 (CH), 41.7 (CH), 49.4 (CH), 50.1 (C-13), 53.9 (NCH_2_), 61.2 (d, *J* = 5.4 Hz, OCH_2_CH_3_), 61.4 (d, *J* = 5.4 Hz, OCH_2_CH_3_), 63.6 (OCH_2_), 110.8 (d, *J* = 9.9 Hz, C-2), 114.9 (d, *J* = 182.5 Hz, C-4), 122.7 (C=CH), 126.0 (2 C, C-3′, and C-5′), 127.9 (2 C, C-2′, and C-6′), 131.4 (d, *J* = 1.8 Hz, C-1), 131.5 (C-1′), 134.4 (d, *J* = 14.4 Hz, C-10), 144.7 and 144.8 (C=CH and C-5), 151.9 (C-4′), 158.9 (C-3), 221.6 (C-17). ^31 ^P NMR *δ* (ppm): 18.2. MS *m*/*z* (%): 634 (100, [M + H]^+^).

### 4.2. Determination of antiproliferative activities

The antiproliferative properties of the newly synthesised triazoles (**11a**,**b**–**15a**,**b**) were determined on a panel of human adherent cancer cell lines of gynaecological origin. MCF-7 and MDA-MB-231 were isolated from breast cancers differing in biochemical background, while A2780 cells were isolated from ovarian cancer. The cancer selectivity of compound **12a** was tested on the non-cancerous mouse embryo fibroblast cell line NIH/3T3. All cell lines were purchased from European Collection of Cell Cultures (ECCAC, Salisbury, UK). Cells were cultivated in minimal essential medium supplemented with 10% foetal bovine serum, 1% non-essential amino acids and an antibiotic–antimycotic mixture. All media and supplements were obtained from Lonza Group Ltd., Basel, Switzerland. Near-confluent cancer cells were seeded onto a 96-well microplate (5000 cells/well) and, after overnight standing, 200 µL new medium, containing the tested compounds at 10 and 30 µM, was added. After incubation for 72 h at 37 °C in humidified air containing 5% CO_2_, the living cells were assayed by the addition of 20 µL of 5 mg/ml 3-(4,5-dimethylthiazol-2-yl)-2,5-diphenyltetrazolium bromide (MTT) solution. MTT was converted by intact mitochondrial reductase and precipitated as purple crystals during a 4-h contact period. The medium was next removed and the precipitated formazan crystals were dissolved in 100 µL of DMSO during a 60-min period of shaking at 37 ^о^C.

Finally, the reduced MTT was assayed at 545 nm, using a microplate reader utilising wells with untreated cells serving as control^27^. In the case of the most active compounds (i.e. higher than 50% growth inhibition at 30 µM), the assays were repeated with a set of dilutions, sigmoidal dose–response curves were fitted to the determined data and the IC_50_ values (the concentration at which the extent of cell proliferation was half that of the untreated control) were calculated by means of GraphPad Prism 4.0 (GraphPad Software, San Diego, CA). All *in vitro* experiments were carried out on two microplates with at least five parallel wells. Stock solutions of the tested substances (10 mM) were prepared in DMSO. The highest DMSO content of the medium (0.3%) did not have any substantial effect on cell proliferation. Cisplatin (Ebewe Pharma GmbH, Unterach, Austria) was used as positive control.

### 4.3. Tubulin polymerisation assay

The effect of brominated triazole (**12a**) on tubulin polymerisation was tested with the HTS-Tubulin Polymerisation Assay Biochem Kit (Bio-Kasztel Ltd., Budapest, Hungary) according to the manufacturer’s recommendations. Briefly, 10 µl of a 0.125 or 0.25 mM solution of the test compound (**12a**) was placed on a prewarmed (37 °C), UV-transparent microplate. About 10 µl 10 µM paclitaxel and 10 µl General Tubulin Buffer were used as positive and negative control, respectively. 100 µl 3.0 mg/ml tubulin in 80 mM PIPES pH 6.9, 2 mM MgCl_2_, 0.5 mM EGTA, 1 mM GTP was added to each sample, and the microplate was immediately placed into a prewarmed (37 °C) UV-spectrophotometer (SpectoStarNano, BMG Labtech, Ortenberg, Germany) to start the recording reaction. A 60-min kinetic measurement protocol was applied to determine the absorbance of the reaction solution per minute at 340 nm. For the evaluation of the experimental data, the maximum reaction rate (*V*_max_: Δabsorbance/min) was calculated. Moving averages of absorbances determined at three consecutive timepoints were calculated and the highest difference between two succeeding moving averages was taken as the *V*_max_ of the tested compound in the tubulin polymerisation reaction. Each sample was prepared in two parallels and the measurements were repeated twice. For statistical evaluation, *V*_max_ data were analysed by the one-way ANOVA test with the Newmann–Keuls post-test by using Prism 4.01 software (GraphPad Software, San Diego, CA).

### 4.4. Computational simulations

#### 4.4.1. Docking studies

In all cases, the Glide package^28^^,^^29^ of the Schrodinger suit^30^ was applied for docking calculations. Dimer structure with a colchicine analogue was cut out from crystal structure (pdb id: 3HKC, www.rcsb.org^31^) for colchicine binding side studies, and a monomer unit in complex with taxol was taken from taxol-stabilized microtubule (pdb id: 5SYF).

The protein preparation wizard^32^ was applied in the Maestro GUI^33^ for the preparation of the downloaded rough crystal structures, and docking grids were prepared first. Each grid was centred to the original crystal ligand position, and default box size was applied. Following the grid generation, single precision (SP) docking was performed with enhanced ligand sampling. In the output, five poses were written out for each ligand.

#### 4.4.2. Molecular dynamics calculations

The MD calculations were carried out with the Desmond^34^^,^^35^ program of the Schrodinger suit. OPLS3e forcefield^36^ in combination with SPC explicit water model was applied in physiological salt concentration. Orthorhombic box with 10 Å buffer size was set up, and single strand 250 ns long NPT MD running was performed at 310 K after the relaxation of the system. The Nose–Hoover^37^ thermostat and Martyna–Tobias–Klein barostat were applied with default relaxation times. The MMGBSA interaction energies were determined by taking 250 snapshots periodically from the MD trajectories and the thermal_mmgbsa.py script of the Desmond program was applied to calculate the binding free energy of a ligand.

## 5. Conclusions

In conclusion, new ring A modified 13α-oestrone derivatives have been synthesised via directed combination of different structural elements. Certain new compounds displayed potent antiproliferative action against human reproductive cancer cell lines. 4-Bromo derivative **12a** exerted submicromolar cell growth-inhibitory action against A2780 cell line. Computational simulations reveal strong interactions of compound **12a** with colchicine and taxoid binding sites of tubulin. Direct effect of compound **12a** on microtubule formation was demonstrated via tubulin polymerisation assay.

## Supplementary Material

Supplemental Material
