# Supplementary material for: Synthesis and evaluation of anticancer activities of 2- or 4-substituted 3-(N-benzyltriazolylmethyl)-13α-oestrone derivatives
Source: J Enzyme Inhib Med Chem. 2020 Oct 29;36(1):58–67. doi: 10.1080/14756366.2020.1838500 (PMC7598997; doi:10.1080/14756366.2020.1838500)
Supplement: Supplemental Material [file IENZ_A_1838500_SM0247.pdf]

SI-Figure 1: RMSD values of C atoms in the common ring part for compound **3** (A-B) and compound **12a** (C,D) as well as RMSD of the peptide backbone in the 15 Å environment of the ligand along the 250ns MD simulation. The environments in A and C were the Colchicine binding site, while in B D we demonstrated the Taxane binding pocket.

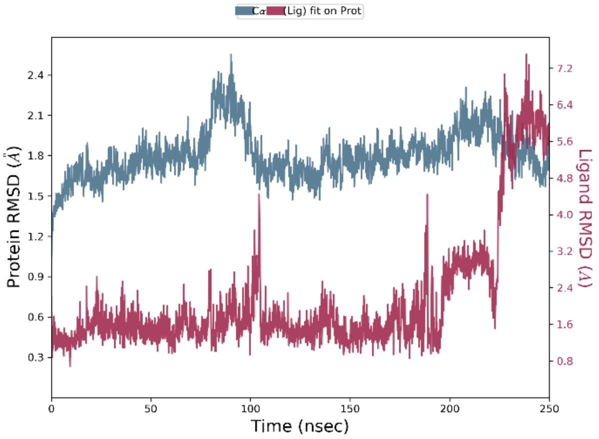

A

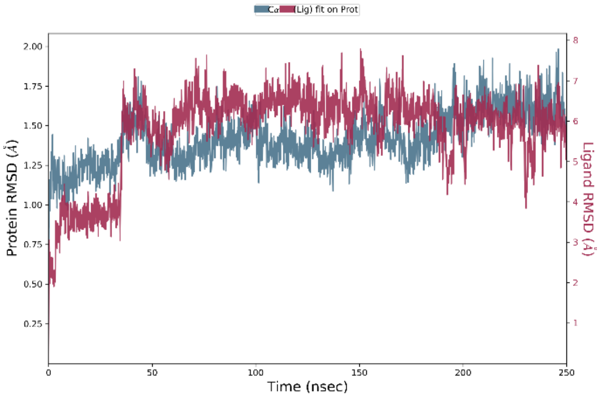

B

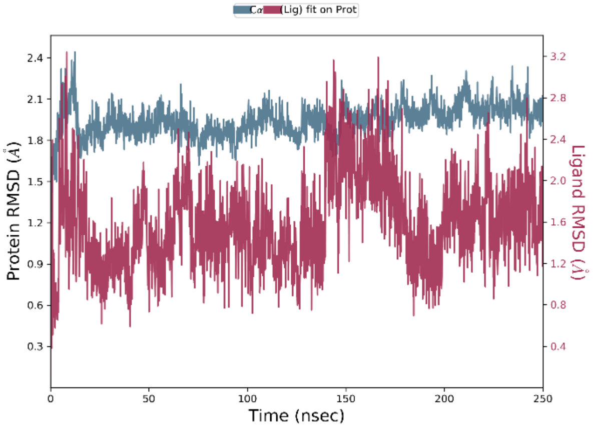

C

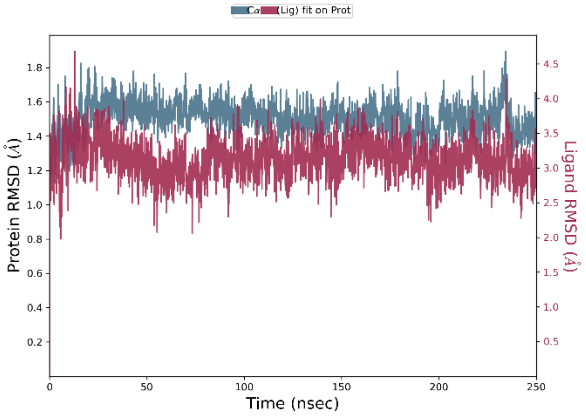

D
